# Pauci Immune crescentic glomerulonephritis in a patient with T-cell lymphoma and argyria

**DOI:** 10.1186/s12882-016-0259-x

**Published:** 2016-05-17

**Authors:** Tamer Rezk, James Penton, Anna Stevenson, Mared Owen-Casey, Mark Little, John Cunningham, Alan D. Salama

**Affiliations:** UCL Centre for Nephrology, Royal Free London NHS Foundation Trust, Rowland Hill Street, London, NW3 2PF UK; Department of Histopathology, Royal Free London NHS Foundation Trust, Pond Street, London, NW3 2QG UK; Trinity Health Kidney Centre, Institute of Molecular Medicine, St James’s Hospital campus, Dublin, D08 W9RT Ireland

**Keywords:** Glomerulonephritis, Silver, Toxicity, Immunosuppression, AKI, Argyria

## Abstract

**Background:**

Silver is a transition metal, toxic when ingested in significant amounts, causing argyria (skin deposition) and argyrosis (eye deposition). It is excreted mainly via the gastrointestinal tract with only small amounts eliminated by the kidneys, and rarely have cases of nephrotoxicity due to silver been reported. Here we present the case of a woman who used colloidal silver as an alternative remedy for a T cell lymphoma, who subsequently developed argyria and a pauci-immune crescentic glomerulonephritis with evidence of extensive glomerular basement membrane silver deposition.

**Case Presentation:**

A 47 year old woman of Indo-Asian descent with a T-cell lymphoma who refused conventional chemotherapy for 18 months but self-medicated with a remedy containing colloidal silver, was admitted with acute dialysis-dependent kidney injury. A kidney biopsy demonstrated a pauci-immune crescentic glomerulonephritis with deposition of silver particles in the mesangium and along the glomerular basement membranes. The patient was treated with intravenous methylprednisolone and intravenous cyclophosphamide and recovered independent renal function.

**Conclusion:**

Chronological evolution of the the pauci-immune glomerulonephritis suggests that a cellular immune-mediated process was induced, potentially mediated by lymphomatous T cells directed at the glomerular basement membrane, following silver deposition. Immunosuppressive therapy improved the situation and allowed cessation of haemodialysis, supporting the hypothesis of an immune-mediated process.

## Background

Silver is a transition metal element with a range of industrial and ornamental uses. Silver also has pharmaceutical applications, primarily as a disinfectant and antimicrobial, and is known to be toxic when ingested in significant amounts. The recognised manifestations of this are irreversible deposition in the eye, causing argyrosis, and in the skin, mucous membranes and internal organs, causing argyria.

Ingested silver is thought to be excreted mainly via the gastrointestinal tract with only small amounts eliminated by the kidneys [1]. Nevertheless some cases of nephrotoxicity due to silver have been reported [2–6]. Here we present the case of a woman who used colloidal silver as a remedy for an un-treated T cell lymphoma and who subsequently developed argyria and a pauci-immune crescentic glomerulonephritis.

## Case presentation

A 47-year-old woman of Indo-Asian decent was admitted to the hospital with a two week history of peripheral oedema, shortness of breath and lethargy, as well as an itchy pigmented rash on her arms, legs and groin of two months’ duration. She reported a large swelling of the right side of her neck, which had been biopsied eighteen months earlier and diagnosed as a T cell lymphoma, for which she had refused conventional chemotherapy. She denied taking any regular prescribed medications, but began using alternative remedies since the appearance of the neck mass.

On examination the rash was raised and hyperpigmented with evidence of excoriation, and the patient had a grey tint to her skin. She had a blood pressure of 164/96 mmHg. The neck mass was firm and non-tender, measured 10cm x 4cm, and had two overlying scars from previous biopsies.

Laboratory results demonstrated a serum creatinine of 600 μmol/l, urea 21.2 mmol/l, haemoglobin 6.3 g/dl, neutrophils 21.9 x 10^9^/l, CRP 118 mg/l, LDH 675 iU/l, and serum bicarbonate 10 mmol/l. Urinalysis confirmed proteinuria and haematuria and urinary protein creatinine ration (UPCR) revealed nephrotic range proteinuria of 515 mg/mmol. Virological tests for HIV, HCV and Hepatitis B sAg were negative. Serological tests for anti-dsDNA, ANA, ANCA and anti-GBM antibodies were all negative, complement levels C3 and C4 were normal.

The patient was commenced on haemodialysis. A CT scan of the chest, abdomen and pelvis confirmed that the neck mass was an enlarged lymph node, with additional axillary and mediastinal lymphadenopathy.

**Fig. 1:**
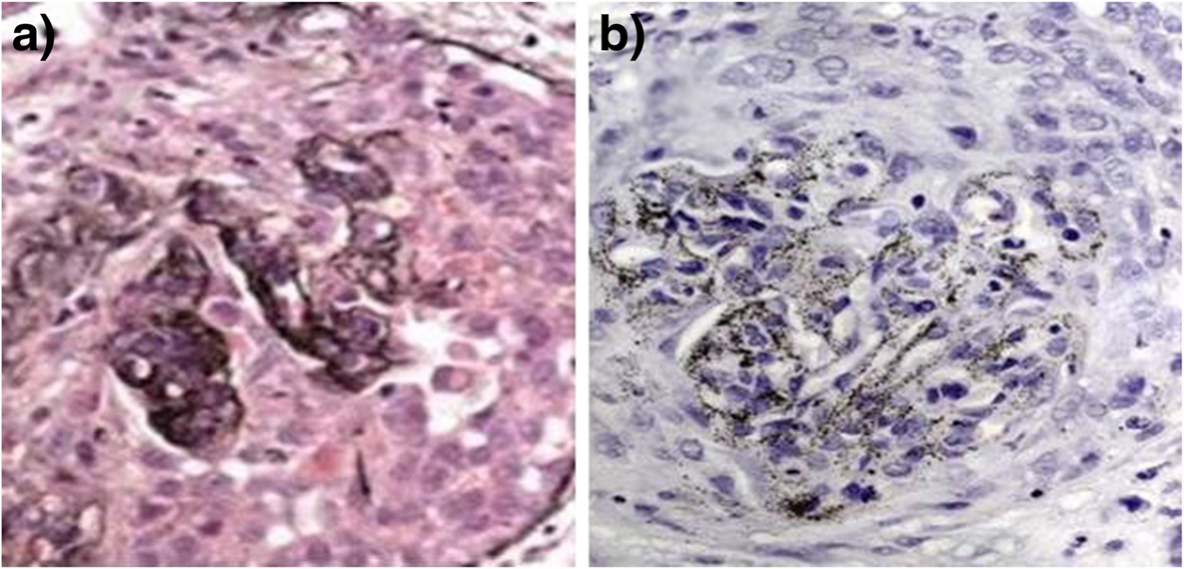
Auramine stain and congo red stain of a single glomerulus demonstrating crescentic change and silver deposition. **a** High magnification of silver-stained renal biopsy showing compression, disruption and destruction of the glomerular basement membrane and **b** demonstrating deposition of dark-staining silver granules (both x600)

**Fig. 2:**
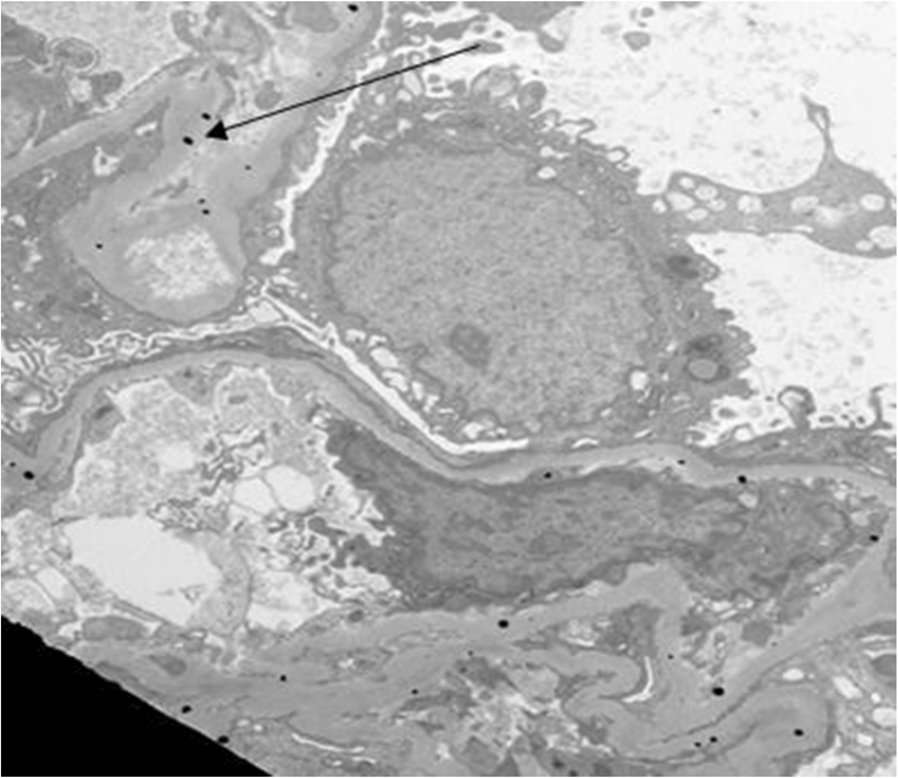
Electron micrograph of a single glomerular capillary loop showing dense dots of sliver scattered within mesangium and glomerular basement membranes (arrows) (magnification x4400)

Biopsies of the kidneys, neck mass and skin lesions were performed. The kidney biopsy revealed pauci-immune crescentic glomerulonephritis, and glomerular tufts with fine, dark, granular material scattered within the mesangium and along the basement membrane (Fig. 1). Electron microscopy confirmed the presence of scattered electron-dense granules along and within the glomerular basement membrane as well as in the mesangium (Fig. 2). This was strikingly similar to the skin biopsy showing scattered fine black granules within the basement membranes of the sweat gland epithelial cells consistent with argyria.

**Fig. 3:**
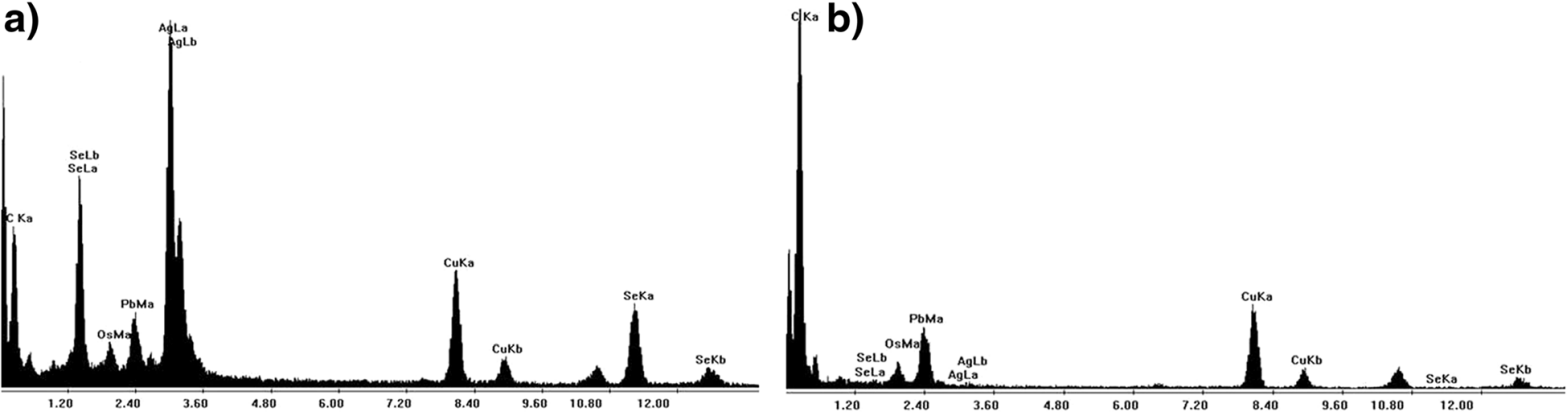
Electron probe microanalysis of **a** granules and **b** background kidney. Silver was detected using energy dispersive analysis of X-rays (EDAX) on the transmission electron microscope. Electron probe microanalysis of background kidney shows a relative absence of silver and selenium atoms with much carbon. Analysis of the granules shows that they contain predominantly silver and some selenium atoms

Electron probe microanalysis demonstrated that these granules contained predominantly silver and some selenium atoms (Fig. 3). The biopsy of the neck mass confirmed a peripheral T-cell lymphoma.

**Fig. 4:**
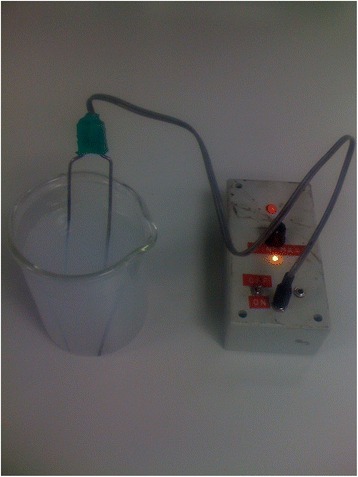
Silver electrode for production of colloidal silver suspension. The device is shown switched on with electrodes placed in a beaker of plain water which has turned cloudy following 15 min of electrolysis

On further questioning the patient admitted using colloidal silver as another treatment for the neck swelling, made by electrolytic solubilisation of a pair of silver electrodes and consumption of the resulting solution (Fig. 4). The patients’ serum silver concentration was strikingly elevated at 127.1 nmol/l (reference value <2.8 nmol/l).

The patient was treated with 3 pulses of intravenous methylprednisolone and intravenous cyclophosphamide, adjusted for age and renal function, every 2-3 weeks, followed by a tapering dose of oral prednisolone. After three weeks, partial recovery of renal function permitted withdrawal of haemodialysis. The patient declined conventional chemotherapy for her lymphoma, but continued pulsed cyclophosphamide to treat her renal impairment. She achieved a nadir creatinine of 288 μmol/l.

## Conclusion

Irreversible silver exposure causing argyria (skin deposition) and argyrosis (eye deposition) is well-recognised. Current evidence for an effect of silver administration on the kidney is limited. Studies of workers occupationally exposed to silver have produced variable results: Rosenman et al. [7] observed a significantly lower creatinine clearance in precious metal manufacturing workers, but could not exclude a contribution of cadmium and solvent exposure, and renal biopsies were not performed. Pifer et al. [8] observed no impairment of creatinine clearance in silver reclamation workers.

A number of case reports of renal impairment following silver exposure have been published. However in several the route of exposure was through the application of topical silver sulfadiazine [2–4], the sulfadiazine component in itself being potentially nephrotoxic, whilst there were also other potentially confounding comorbidities such as diabetes, hypertension [2] and severe burns [3]. Watanabe et al. [5] reported an instance of nephrotic syndrome and membranous glomerulonephropathy in a patient who had had argyria for 8 years. More recently, Mayr et al. [6] published a case of renal impairment in a hypertensive patient who had been ingesting colloidal silver. His renal biopsy appearances were similar in part, with deposited silver particles along the glomerular basement membrane, but unlike our case glomerular damage was limited to hypertensive and ischaemic glomerulosclerosis.

Crescentic glomerulonephritis results from disruption of the glomerular basement membrane (GBM) which may be induced by immune complexes or by cellular mediators such as proteinases and reactive oxygen species. Examples of damage to the GBM initiating a pathological immune response by exposure of an immune neo-epitope and subsequent crescentic glomerulonephritis include membranous glomerulonephritis [9] and lithotripsy [10] in association with anti-GBM antibodies. ANCA positive pauci-immune glomerulonephritis has been previously described in a patient with intravascular large B cell lymphoma who presented with livedo reticularis and systemic symptoms of fever and lethargy however in this case renal excretory function was preserved [11]. Hamidou et al. described 2 cases of ANCA vasculitis in the context of chronic lymphocytic leukaemia and T cell lymphoma with both cutaneous vasculitis and a pulmonary renal syndrome [12]. However, our patient had no autoantibodies, and no deposited immunoproteins. Our case is unique in that it represents an ANCA negative pauci-immune crescentic glomerulonephritis with evidence of intense silver deposition along the GBM in a patient with T cell lymphoma.

Although most cases of pauci-immune GN are associated with ANCA, between 5–10 % cases are ANCA negative. The pathogenesis in these cases may be related to other autoantibodies (such as anti-endothelial antibodies) or to direct leukocyte-induced GBM damage via soluble mediators, which in this case may have been due to a combination of T cell lymphoma and argyria. We demonstrate that immunosuppressive therapy improved the situation and allowed cessation of haemodialysis, supporting a hypothesis of an immune-mediated process most likely directed towards the glomerular basement membrane associated with silver deposition.

### Ethics and consent to participate

All relevant ethics for the case report and its publication were obtained.

### Consent to publish

Written informed consent was obtained from the patient for publication of this case report and any accompanying images. A copy of the written consent is available for review by the editor of this journal.

## Abbreviations

HIV: Human immunodeficiency virus
HCV: Hepatitis C virus
Hepatitis B sAg: Hepatitis B surface antigen
anti- dsDNA: Anti double stranded DNA
ANA: Anti-nuclear antibodies
ANCA: Anti neutrophilic cytoplasmic antibody
anti-GBM: Anti glomerular basement membrane antibodies
C3: Complement 3
C4: Complement 4
CT: Computed tomography
GN: Glomerulonephritis
